# Healthcare Transformation in Singapore With Artificial Intelligence

**DOI:** 10.3389/fdgth.2020.592121

**Published:** 2020-11-17

**Authors:** Siqi Liu, Qianwen Stephanie Ko, Kun Qiang Amos Heng, Kee Yuan Ngiam, Mengling Feng

**Affiliations:** ^1^National University of Singapore Graduate School for Integrative Sciences and Engineering, National University of Singapore, Singapore, Singapore; ^2^Saw Swee Hock School of Public Health, National University of Singapore and National University Health System, Singapore, Singapore; ^3^Division of Advanced Internal Medicine, National University Hospital, Singapore, Singapore; ^4^Group Chief Technology Office, National University Health System Singapore, Singapore, Singapore

**Keywords:** healthcare datathon, datathon model, healthcare transformation, artificial intelligence, machine learning

## Introduction

Singapore's healthcare system is in the process of transformation. From country-wide vaccination programmes started during Singapore's independence focusing on infectious diseases (1), to the establishment of polyclinics, general hospitals, and specialist centers to address chronic disease management, to the current COVID-19 pandemic (2), healthcare challenges are constantly evolving. Although Singapore has excellent healthcare outcomes with an average life expectancy increased over the years (3), we face the combined challenge of rising healthcare costs, aging population with an increasing burden of chronic diseases and shortage of healthcare professionals. Diabetes alone is projected to affect more than 1 million people by 2050, more than double compared to 2014 (4).

To overcome these challenges, Singapore needs to transform the way it delivers care. The Ministry of Health Singapore (MOH) has framed the healthcare transformation using the “3 Beyonds” strategy—“Beyond Healthcare to Health, Beyond Hospital to Community, Beyond Quality to Value” (5). “Beyond Healthcare to Health” involves strategies to promote the early detection of chronic diseases and prevent diseases with healthy lifestyles. “Beyond Hospital to Community” drives a shift in care from the hospital to primary care and then to the home, enabling aging in place at home. “Beyond Quality to Value” strives for improved efficiency to achieve better outcomes while lowering costs and reducing waste. The explosive increase in the amounts of healthcare data, along with the improved performance of Artificial Intelligence (AI) is poised as a transformational force in healthcare.

First of all, AI tools have demonstrated the capability to enable the creation and delivery of better management services to deal with chronic diseases. Many research groups have investigated the application of AI to control blood glucose for diabetes mellitus (DM) (6–9). Many of them have been tested using virtual patients or simulations. For example, Mauseth et al. (6) designed an AI-based controller, namely fuzzy logic controller (FLC) to personalize glycemic control by determining the dosing of insulin on artificial pancreas. Daskalaki et al. (7) presented an adaptive, patient-specific blood glucose control strategy based on the another AI approach called reinforcement learning, and tested their approach on 28 virtual type-1 DM patients. Their results suggested that the AI approach could improve nocturnal blood glucose control without increasing the risk of hypoglycaemia.

Secondly, AI could also be used as a digital health coaching tool that help patients to manage their conditions at home. AI has empowered chatbots to provide virtual assistance to patients and answer simple questions about their condition. For example, Stein et al. (10) developed a pure AI-driven fully automated conversational health coaching mobile application for obese adults. Their results showed that the AI application could help the participants to achieve weight loss comparable to in-person lifestyle interventions. Other researchers have developed “AI doctors” that provide health advice directly to patients with common symptoms, freeing up primary care access for more complex care (11). A survey conducted on 800 European and American patients with atrial fibrillation, Type 2 diabetes or breast cancer found that while many patients did not want their doctors replaced by AI, most of them were happy with a round-the-clock virtual assistance from an AI agent (12).

Last but not least, AI could help to improve accuracy and efficiency of disease diagnosis. The AI-powered algorithms for diagnosing disease is now outperforming physicians in detecting skin cancer (12), breast cancer (13), and brain tumors (14). Besides, AI can also help to quickly process large volumes of both unstructured and structured data including past medical history, continuous physiologic signals (such as vital signs), laboratory results, genomics data, imaging reports, and many others. As a result, the physicians can better focus on more complex cases and improve the efficiency of diagnosis.

AI-based approaches could empower individuals to take charge of their health as well as enhance the quality and accessibility of care. It would be a powerful force for the healthcare transformation if it is implemented wisely. Poorly implemented AI applications in healthcare would lead to extra burdens and increased risks for both patients and physicians. Therefore, it is extremely important to make sure all the AI-based approaches are validated by clinical proof-of-concepts (POC) before entering large clinical trials and implementing to clinical workspaces. Hence, we propose “healthcare datathon” as a testbed for clinical POC and a pilot for the development of strategies to deal with real clinical problems.

## Healthcare Datathon

The word datathon is a portmanteau for a data hackathon, which is originated from technology companies as internal events for engineers and computer scientists to collaborate, brainstorm, and build innovative solutions to challenging, company-wide problems in a concentrated period of time (15). Thereafter, the datathon model has been adapted to other domains to address problems with broader interests rather than company-exclusive problems. In particular, a healthcare datathon bring together interdisciplinary teams of students and professionals from both technical and healthcare domain to collaborate, brainstorm, and build solutions to unmet clinical needs.

The goal of a healthcare datathon is to enhance collaboration among participants from various backgrounds to produce clinically relevant research that relies on sound statistical rigor and adequate data samples. It features an iterative process of idea elaboration and group learning among individuals with different types and levels of expertise in a mutually supportive environment.

**Figure 1:**
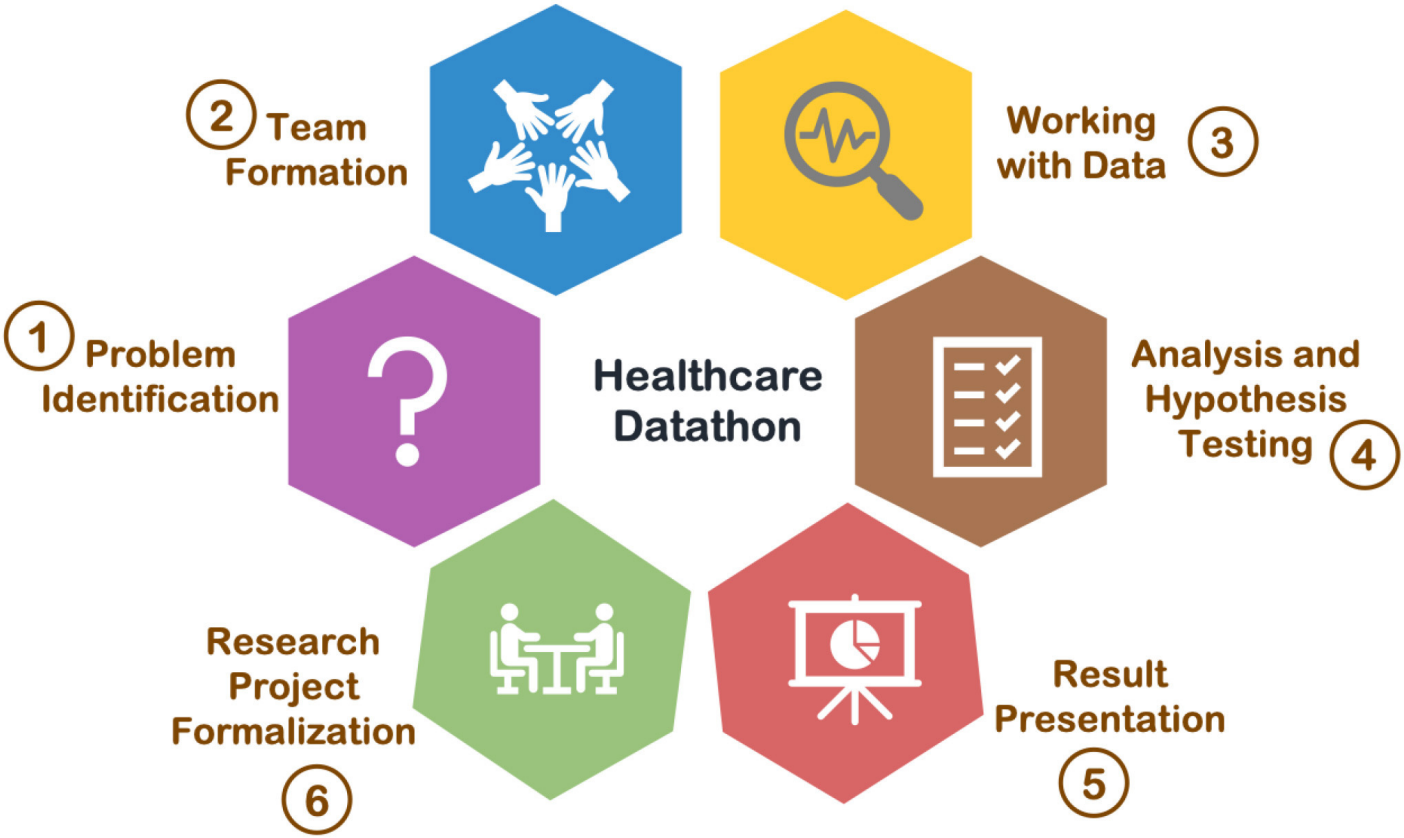
Process of a healthcare datathon.

Healthcare datathon is usually an annual event which spans 2–3 days and follows a 6-stage process illustrated in Figure 1. (1) Problem identification: clinical needs/questions are presented to participants by healthcare professionals. (2) Team formation: interdisciplinary teams are formed around the clinical question by healthcare professionals, computer scientists, statisticians, and other data enthusiasts. (3) Working with data: data extraction, cleaning, and processing are performed by team members from technical background. All teams share a common clinical database. (4) Analysis and hypothesis testing: teams brainstorm the solutions to the clinical question and conduct analysis to verify their hypothesis. (5) Result presentation: teams present their ideas and results to judges and the all the participants. (6) Research project formalization: if a clinical solution or a hypothesis is validated through stage (1)–(5), teams can formalize the clinical question to a research project, and continue to work on the project after the healthcare datathon.

A real practice of healthcare datathon, named, “Healthcare AI Datathon and Expo,” was organized in Singapore on July 6–8, 2019 (event videos and the participants information and can be referenced in the Supplementary Material). The clinical questions were proposed by physicians in a local hospital and were later revised by clinical researchers to verify their relevance and feasibility of developing a solution. In total, 17 questions were selected and categorized into two tracks: Critical Care and Medical Imaging (Table 1). Following the 6-stage process of datathon, clinical leads first pitched their clinical questions to rest of the participants and then were instructed to form teams on their own. All participating teams were then given 2 days to solve their clinical questions and were mentored by data experts and clinical professionals. At the end of the event, all 17 projects were completed within the event timeline. Teams presented their results to a judging panel of data scientists and clinical experts, who selected the champion projects and provided feedbacks and suggestions for future work to individual teams. In the last stage, teams with promising results would continue their project, and transform it into academic research.

**Table 1:** Singapore AI healthcare datathon and expo 2019 project list.

**Track**	**Team**	**Clinical question**
Critical care	1	Predicting neurological recovery in traumatic brain injury in the ICU^a^
	2	Define the optimal blood pressure and its range for critically ill patients
	3	Development of new scoring system for sepsis
	4	Causal inference of changeover month on patient outcomes
	5	Prediction of utility and futility of renal replacement therapy in AKI patients
	6	Risk prediction for intra-cranial hemorrhage
	7	Longitudinal trends of high risk asthma
	8	A predictive model for ventilator associated events
	9	Individualized duration of dual antiplatelet therapy after percutaneous coronary intervention
	10	Impact of drug-drug combinations on length of mechanical ventilation for critically ill patients
	11	Predicting chronic kidney disease progression of ICU patients from lab results
	12	Prognostic factors associated with mortality among ICU patients with clostridium difficile infection
	13	Prediction of 30-day readmission
	14	Prediction of night-shift deterioration
Medical imaging	15	Automated diagnosis of chest x-rays with deep learning
	16	Skin lesion segmentation for melanoma detection
	17	Classification and segmentation of pneumothorax with chest x-rays

a *ICU, intensive care unit*.

## Discussion

The healthcare datathon that organized in Singapore in 2019 brought together a diverse group of participants with wide-ranging domains of expertise. The event attracted more than 200 participants from over 8 countries, including healthcare workers from local hospitals, academic researchers and students from local and overseas universities, and various data scientists from industry. The majority of participants (56%) had backgrounds in data science, others were healthcare professionals (28%), and graduates (5%) and undergraduate (11%) students from science and engineering faculties in local universities. With media exposure and industry-wise collaborations, we observed an increased number of participants with a diversity of backgrounds compared to the past events. The clinical questions in datathon can be roughly classified into 4 types: the prediction of disease progressions (*n* = 9, 53%); analysis of the treatments' impact on patient outcomes (*n* = 3, 18%); training of automated AI tool for disease diagnosis (*n* = 3, 18%); and development of new clinical definition/guideline for disease management (*n* = 2, 12%). Among all the teams in healthcare datathon over the years, we found that on average, 10% of teams remained active after the event. They were able to formalize their clinical problems into research projects and produce impactful publications (16–19). For example, van den Boom et al. (16) were a group of researchers who participated in healthcare datathon 2018 in Singapore and formed a research team to study the optimal oxygen saturation target for critically ill patients. Upon the completion of datathon, they identified a potential optimal ranges of SpO_2_ among patients requiring oxygenation therapy. Working collaboratively among the health professionals and data scientists in the group, van den Boom et al. managed to validate their proposed optimal range in two large public electronic medical records databases (20, 21) and published their findings. Their research project were successfully conducted and translated into clinical knowledge. Later, their findings were studied and referenced by many other researchers in the field (22–24), and had potential to impact real clinical practice during the pandemic by guiding the respiratory support for critically ill patients with COVID-19 (22, 24).

Healthcare datathon can benefit AI-based clinical research in several ways. First of all, healthcare datathon is served as a testbed and a clinical POC to validate ideas and hypothesis. By analyzing the real-world clinical data, healthcare datathon encourages effective discussion between healthcare professionals and data scientists to validate and prototype clinical problems with solutions over the course of the event. Secondly, healthcare datathon is a collaborative event that assemble interdisciplinary teams of experts with medical, engineering, business background, so that the team could develop interdisciplinary solution to the unmet clinical needs while drawing upon best practices from the technology industry. Thirdly, healthcare datathon provides a great opportunity for participants to exchange ideas and gain valuable research experience. Some participants may also find datathon beneficial for their own research by getting inspiration from projects presented by other teams. Last but not the least, healthcare datathon is an effective platform to build teams with common research interest and diverse expertise. Therefore, clinical problems can be smoothly formalized to research projects, and translate to clinical knowledge.

## Summary Findings and Conclusion

- Singapore's healthcare landscape is evolving toward holistic, longitudinal, preventive, and individualized care.
- Artificial Intelligence (AI) has the potential to help in individualizing and improving prevention, diagnosis, and treatment for healthcare transformation.
- In particular, the “healthcare datathon” can be used as an effective model to testbed clinical POC with real clinical problems.

The healthcare datathon was an effective platform to bring together people with various backgrounds and expertise. For data scientists, the event provided them with an opportunity to work with clinicians, understand the clinical questions in-depth and apply their skills on real health data. For physicians, the datathon afforded them access to both data and analytic expertise. The event demonstrated an effective method of promoting the cross-discipline collaboration that is needed to transform the healthcare culture in Singapore.

## Author Contributions

SL, KN, and MF contributed to the initiation and the discussion of the research topic. SL, QK, KH, KN, and MF contributed to the analysis of the contents and to the writing of the manuscript. All authors contributed to the article and approved the submitted version.

## Conflict of Interest

The authors declare that the research was conducted in the absence of any commercial or financial relationships that could be construed as a potential conflict of interest.
