# Supplementary material for: Healthcare Transformation in Singapore With Artificial Intelligence
Source: Front Digit Health. 2020 Nov 17;2:592121. doi: 10.3389/fdgth.2020.592121 (PMC8521861; doi:10.3389/fdgth.2020.592121)
Supplement: Supplementary file 1 [file Data_Sheet_1.docx]

Supplementary Material

# Supplementary data

# Supplementary Video 1. Singapore Healthcare Datathon 2018 event video.

# [
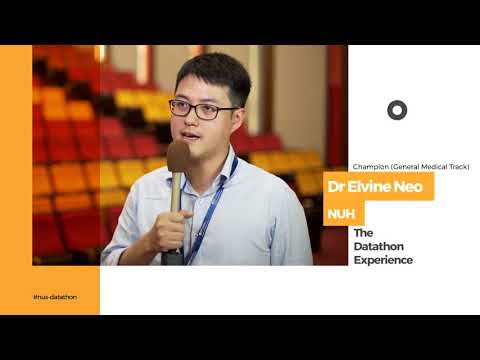
](https://www.youtube.com/embed/oDL3JoCAo-0?feature=oembed)

# Supplementary Video 2. Singapore Healthcare Datathon 2018 participants interview Part I[
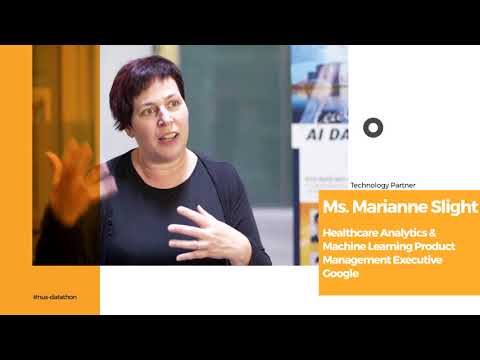
](https://www.youtube.com/embed/kj9xWZcxTJ8?feature=oembed)

**Supplementary Video 3.** Singapore Healthcare Datathon 2018 participants interview Part II

[
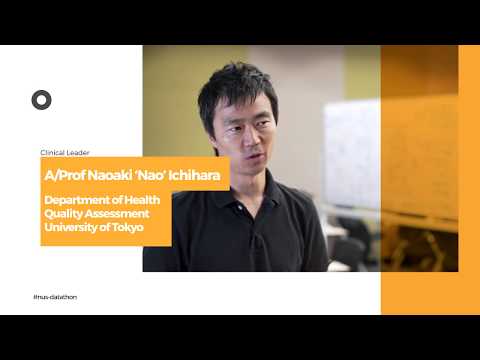
](https://www.youtube.com/embed/eCkNKSCV9uQ?feature=oembed)

**Supplementary Video 4.** Singapore Healthcare Datathon 2019 event video.

**[
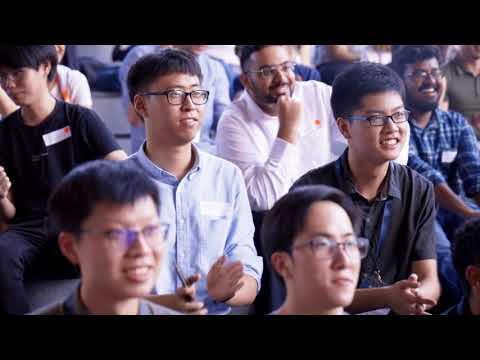
](https://www.youtube.com/embed/zpm4u-psyfg?feature=oembed)**
